# Potential Diets to Improve Mitochondrial Activity in Amyotrophic Lateral Sclerosis

**DOI:** 10.3390/diseases10040117

**Published:** 2022-12-01

**Authors:** Sayuri Yoshikawa, Kurumi Taniguchi, Haruka Sawamura, Yuka Ikeda, Ai Tsuji, Satoru Matsuda

**Affiliations:** Department of Food Science and Nutrition, Nara Women’s University, Kita-Uoya Nishimachi, Nara 630-8506, Japan

**Keywords:** ALS, mitophagy, ROS, AMPK, mTOR, mTORC1, natural product

## Abstract

Amyotrophic lateral sclerosis (ALS) is an incurable neurodegenerative disease, the pathogenesis of which is based on alternations in the mitochondria of motor neurons, causing their progressive death. A growing body of evidence shows that more efficient mitophagy could prevent and/or treat this disorder by suppressing mitochondrial dysfunction-induced oxidative stress and inflammation. Mitophagy has been considered one of the main mechanisms responsible for mitochondrial quality control. Since ALS is characterized by enormous oxidative stress, several edible phytochemicals that can activate mitophagy to remove damaged mitochondria could be considered a promising option to treat ALS by providing neuroprotection. Therefore, it is of great significance to explore the mechanisms of mitophagy in ALS and to understand the effects and/or molecular mechanisms of phytochemical action, which could translate into a treatment for neurodegenerative diseases, including ALS.

## 1. Introduction

Mitochondrial activity and the generation of reactive oxygen species (ROS) are important for cell proliferation, survival, and/or differentiation [1]. In addition, many diseases, such as cardiac failure, cancer, and age-related pathological conditions, have been related to altered mitochondrial function [2]. For example, the energy deficiency resulting from local hypoxia during an ischemic heart attack leads to mitochondrial dysfunction, which could have arrhythmogenic consequences and lead to sudden cardiac death [3]. The significance of mitochondria has been emphasized in a variety of neurodegenerative diseases, including amyotrophic lateral sclerosis (ALS). ALS is an incorrigible neurodegenerative disease whose etiology is based on the progressive death of motor neurons [4]. At present, ALS has no effective therapy. A better understanding of the mitochondrial regulating pathways may raise several promising neuroprotection perspectives in the effective treatment of ALS. Furthermore, the development of new treatments would be useful not only for the mitochondrial disorders of ALS but also for the wide spectrum of age-related neurodegenerative diseases.

The mitochondrion is the main place for adenosine triphosphate (ATP) synthesis, fatty acid β-oxidation, and ROS production [5]. Mitochondrial function and ATP production are crucial for neuronal cell survival and excitability [6]. However, mitochondrial dysfunction leads to the overproduction of ROS and neuronal apoptosis, which is closely related to neurodegenerative diseases [6]. To slow down the progression of the pathology of ALS, the high oxidative stress and mitochondrial activity of neurons should at least be improved. Mitophagy, which is a specific kind of autophagy, can selectively degrade damaged mitochondria to reduce mitochondrial dysfunction and maintain mitochondrial function. Thus, mutations in the genes that encode factors essential for mitophagy may result in the impairment of this process, which can lead to neurodegenerative conditions such as ALS [7]. For example, hypoxia is a common factor in several disease conditions, such as inflammation, which can lead to a depletion of oxygen and, eventually, through the production of ROS, directly to an alteration of intracellular proteins, lipids, or DNA [8]. Therefore, the control of the mitochondrial ROS level is of key relevance for maintaining cellular homeostasis [9]. The conserved pathway of mitophagy is required to prevent and/or counteract the pathogenic actions that may lead to neurodegeneration. Here, we outline the mechanisms leading to mitochondrial dysfunction in ALS and discuss the potential of targeting mitophagy with several phytochemicals for the possible treatment of ALS, with a view to providing a direction for finding certain phytochemicals that can target mitophagy to prevent and/or treat ALS.

## 2. Mitochondrial Dysfunction Involved in the Pathogenesis of ALS

Mitochondria are pretty vigorous organelles whose number and activity can be adjusted to the changes in cellular energy metabolism by regulating their biogenesis, fusion/fission events, and removing damaged ones [5]. The most common reasons for mitochondrial dysfunction are hypoxia and the overproduction of ROS. In particular, oxidative stress caused by numerous inflammations is related to the development of many neurological complications [10]. Excessive ROS also induce oxidative stress and apoptosis in cells [11]. Typically, in cells, ROS are generated in the mitochondrial respiratory chain. Accordingly, mitochondrial dysfunction could also lead to neuronal cell death and/or apoptosis [12], which is associated with neurological complications, including Alzheimer’s disease, Parkinson’s disease, and Huntington’s disease [13]. These oxidative stresses that include oxygen radicals could be intercepted [14]. In various neurodegenerative diseases such as ALS, several defects in mitochondrial function leading to oxidative stresses have been identified in underlying relations [15]. Indeed, this emphasizes the importance of healthy mitochondria for the maintenance of healthy neuronal functions.

Under physiological conditions, ROS could work as regulators of the mechanism for maintaining cellular redox homeostasis [16]. During oxidative phosphorylation, mitochondria could produce a superoxide anion by-product, which may be further changed into ROS [17]. Mitochondrial ROS consist of superoxide, hydrogen peroxide, and hydroxyl, which can modify lipids, proteins, and DNA, resulting in mitochondrial dysfunction and/or neuronal cell death [18]. Accumulation of damaged mitochondria and the overproduction of ROS will strengthen each other, which may finally lead to severe mitochondrial dysfunction. Mitochondrial ROS are also regulators of a cellular redox environment linked to cellular metabolic balance [19]. Accordingly, hindering too much production of ROS is considered an effective way to prevent oxidative damage to cells, including the neuron [20]. It may be indispensable to explore the roles of mitochondrial ROS in ALS. Antioxidants are the first safety to clear ROS [21]. In addition, there are various systems to withstand ROS-induced oxidative stresses, based on superoxide dismutase, catalase, and/or glutathione peroxidases. Considering the crucial role of mitochondria in cellular homeostasis, monitoring the quality of mitochondria may be important for avoiding neurodegenerative diseases including ALS [22]. In fact, functional defects in and altered morphology of mitochondria have been found in the spinal motor neurons of ALS patients [23]. Likewise, mislocalization and aggregation of mitochondria have been detected in the motor neurons of ALS patients. These observations suggest that the dysfunction of mitochondria may be a regular feature of ALS [24]. Therefore, mitochondrial quality control in many forms of molecular and/or cellular levels should be performed to prevent neurodegenerative diseases, including ALS.

Mitophagy, a mitochondrial quality control mechanism, selectively removes dysfunctional mitochondria to preserve mitochondrial function and maintain cellular homeostasis. In this process, impaired mitochondria are trapped and surrounded by autophagic membranes and further delivered to lysosomes, where they are degraded. It is well-known that the clearance mechanism of damaged mitochondria can be a potent therapeutic strategy in cases of increased oxidative stresses [25]. Some physiologic or chemical faults in mitochondria may trigger mitophagy by disrupting the mitochondrial inner membrane, possibly with the involvement of ROS. Mitophagy has been proven to be related to the development of various diseases, including ALS [26]. For example, treatment with progesterone can prolong the survival time in a mouse model of ALS, which might be associated with enhanced autophagy in the spinal cord [27]. On the contrary, autophagy induction could accelerate the progression of ALS, most likely through the excessive mitochondrial clearance in motor neurons [28]. The balance between synthesis and degradation of mitochondria may be essential for maintaining mitochondrial and/or cellular homeostasis, and the modulation of mitophagy represents a promising therapeutic intervention.

## 3. Mitochondrial Quality Control by Mitophagy in ALS

**Figure 1:**
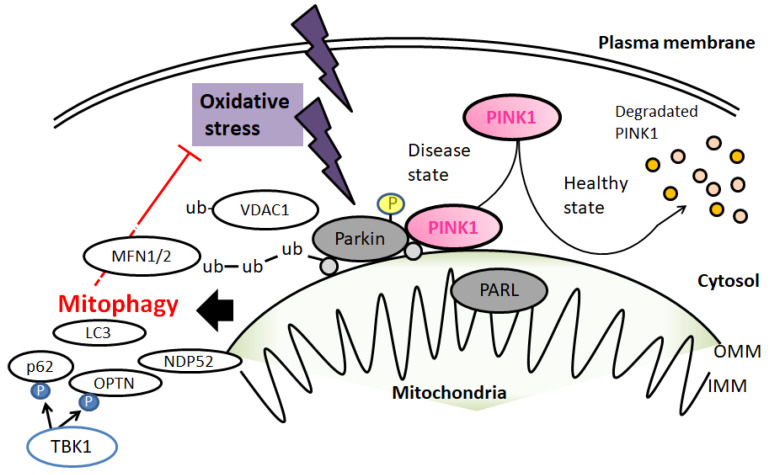
A hypothetical schematic representation and overview of PINK1, Parkin, and related molecules in a regulatory pathway for mitophagy. Under a healthy and steady state of cells, PINK1 is degraded within the surface of mitochondria. This may be inhibited by mitochondrial damage due to various oxidative stresses, resulting in PINK1 and Parkin accumulation in the outer membrane of mitochondria (OMM). Then, PINK1 phosphorylates ubiquitin to activate Parkin ubiquitin ligase activity. In addition, Parkin is also assumed to be phosphorylated and ubiquitinated, resulting in the induction of mitophagy. Note that some critical pathways have been omitted for clarity. OMM: outer mitochondrial membrane; IMM: inner mitochondrial membrane.

Mitochondrial dysfunction is considered an important cause in the pathogenesis of neurodegenerative diseases, which could be inhibited through mitophagy to retain the healthy functioning of the mitochondria. To put it simply, dysfunctional mitochondria could be separated from healthy ones and removed through mitophagy. In details of the molecular mechanisms, damaging mitochondria leads to the accumulation of PTEN-induced kinase 1 (PINK1) in the outer membrane of the mitochondria, where it recruits E3 ubiquitin ligase Parkin that can activate the removal of mitochondria by autophagosomes [29]. In general, PINK1 is located in the outer mitochondrial membrane, but PINK1 cannot be detected in healthy mitochondria (Figure 1). Consequently, PINK1 and Parkin are recruited to the outer mitochondrial membrane for the removal of impaired mitochondria [30], which in turn ubiquitinates mitochondrial surface proteins [31]. Therefore, PINK1 and Parkin are two critical mediators regulating mitophagy in mammalian cells [32] (Figure 1).

There are two major mechanisms involved in Parkin-dependent mitophagy. Parkin can promote the ubiquitination of the mitochondrial fusion proteins mitofusin 1 (MFN1) and mitofusin 2 (MFN2), resulting in their degradation, which leads to mitochondrial fission. This contributes to the separation of dysfunctional mitochondria from the healthy system and allows dysfunctional mitochondria to be surrounded by autophagosome membranes and degraded in lysosomes [33]. Also, Parkin-mediated ubiquitination of mitochondrial outer membrane protein voltage-dependent anion channel 1 (VDAC1) promotes the recognition of VDAC1 using autophagy receptors such as histone deacetylase 6 (HDAC6) [34,35]. Subsequently, PINK1, located in the outer mitochondrial membrane, recruits autophagy receptors, including p62, nuclear dot protein 52 (NDP52), and optineurin (OPTN), which can bind to an autophagosome-membrane-inserted LC3 protein to combine the dysfunctional mitochondria and autophagosomes. Finally, dysfunctional mitochondria are degraded in the lysosome [34] (Figure 1). To further activate mitophagy, PINK1 phosphorylates the ubiquitin of ubiquitin chains attached to mitochondrial proteins and the ubiquitin-like domain of Parkin to facilitate Parkin localization from the cytosol to the outer mitochondrial membrane of the impaired mitochondria [35,36]. Some ALS-associated gene-encoding proteins may play crucial roles in mitophagy. For example, mutant p62 shows a lower affinity for LC3, reducing the efficiency of autophagy [37]. It has been shown that mutations in gene-encoding TANK-binding kinase 1 (TBK1), which phosphorylates and regulates proteins like p62 and OPTN, result in impaired autophagy and contribute to ALS’s pathology [38]. Lysosomal dysfunction has been found in cells with ALS, while lysosomal deficits accompanied by impaired autophagy may occur gradually in a mouse model of ALS [39].

**Figure 2:**
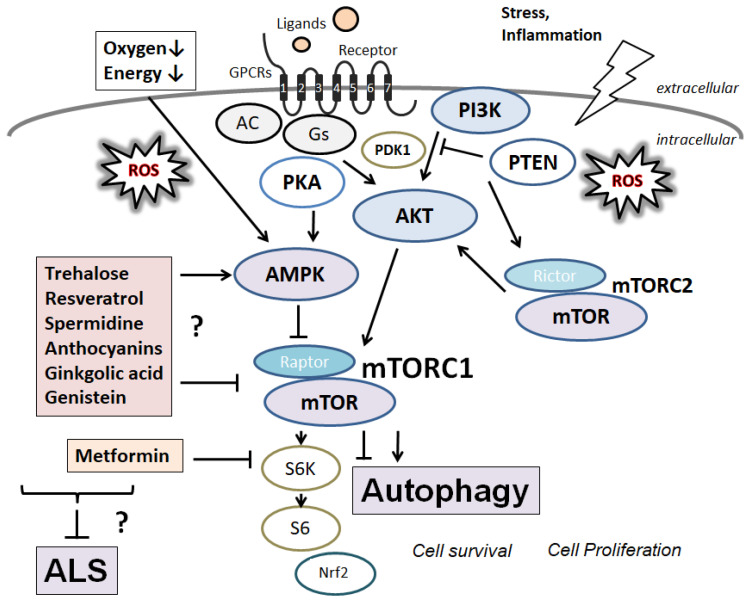
Selected signaling pathways involved in the induction of autophagy with indication of the sites of action of several natural compounds affecting them. Several modulator molecules linked to the PI3 K/AKT/mTOR/mTORC1 signaling pathway are demonstrated. Example compounds from natural sources known to act on the AMPK/mTOR and/or autophagy signaling are also shown. An arrowhead means stimulation, whereas a hammerhead represents inhibition. Note that some critical events, such as immune activation and/or antioxidant feedback, have been omitted for clarity. Abbreviations: AMPK, adenosine monophosphate-activated protein kinase; mTOR, mammalian/mechanistic target of rapamycin; PI3 K, phosphoinositide-3 kinase; PKA, protein kinase A; PTEN, phosphatase and tensin homolog deleted on chromosome 10; mTORC, mechanistic/mammalian target of rapamycin complex; ALS, Amyotrophic Lateral Sclerosis.

Rapamycin, a commonly used immunosuppressive drug, has been shown to increase the survivability of ALS-affected motor neurons [40]. Rapamycin can induce autophagy by inhibiting the mechanistic/mammalian target of the rapamycin (mTOR) pathway [41]. In general, the mTOR pathway plays a key role in the proliferation, survival, and differentiation of various cells. Interestingly, the downmodulation of mTOR activity could increase longevity [42]. Metformin can downregulate the phosphorylation level of S6 kinase, which is a key signaling kinase downstream of the mTOR (Figure 2). Also, metformin can be used to treat a variety of aging-related metabolic disorders, like cardiovascular disease or cognitive decline [43]. The mTOR is involved in two types of protein complexes, mTOR complex 1 (mTORC1) and mTOR complex 2 (mTORC2), albeit with diverse functions [44]. The mTORC1 regularly obstructs autophagy by integrating the upstream signals from PI3 K and/or AKT. The other mTORC2 might not be closed with the regulation of autophagy [45]. However, mTOR could form two complexes, mTORC1, and mTORC2, probably with crosstalk between them. The activated PI3 K could trigger the AKT with 3-phosphoinositide-dependent protein kinase-1 (PDK1) [46]. Then the activated AKT further phosphorylates the tuberous sclerosis protein 2 (TSC2) and obstructs its interplay with TSC1, ultimately resulting in mTORC1 activation [47]. Adenosine monophosphate (AMP)-activated protein kinase (AMPK) also regulates the mTORC1 and serves as an energy sensor [48]. AMPK is stimulated by a decline in ATP concentration during ischemia that raises the ratio of AMP to ATP [49]. Interestingly, the longevity-enhancing effects of metformin mimic the effect of the stimulation of AMPK [50]. Therefore, AMPK-mTOR signaling pathways specifically regulate cellular homeostasis, including autophagy, cell proliferation, differentiation, and energy metabolism [51]. Modulation of autophagy has been shown to be a potential therapeutic target in neurodegeneration [52]. For example, treatment with n-butylidenephthalide could prolong the survival of ALS mice by abrogating autophagy [53]. Accordingly, there is a persistent reason to evaluate whether other agents can stimulate or inhibit autophagy and have a beneficial effect by reducing ALS pathogenesis.

## 4. Beneficial Components from Natural Products for the Treatment of ALS

Increasing evidence emphasizes the beneficial role of naturally derived compounds from different natural sources for the prevention and/or treatment of human diseases, including ALS [54]. Currently, several compounds with antioxidant activity and neuroprotective effects are potential alternative therapies for neurological complications due to their unique therapeutic properties and considerable safety [55]. In addition, there is a variety of natural compounds known for autophagy-modulating properties as well as for controlling oxidative stress and/or active oxygen [56,57].

Trehalose is a natural saccharide that can modify autophagy [58], triggering transient lysosomal damage [59]. Induction of autophagy by trehalose may result in a significant reduction in the number of lysosomes in peripheral blood cells of sporadic ALS, suggesting that trehalose could represent an energetic treatment for ALS patients [60]. Trehalose’s mechanisms of action may involve the inhibition of glucose transporters leading to AMPK activation, which could affect autophagy [61]. Consequently, trehalose could act as a weak inhibitor of the lysosome via the inactivation of mTORC1 [62]. Resveratrol, a natural polyphenolic compound, may indicate many beneficial effects. For example, resveratrol has neuroprotective properties against various neurological disorders [63] through the activation of the AMPK autophagy signaling pathway [64]. Resveratrol could reduce mTORC1 signaling for the activation of autophagy [65]. In addition, resveratrol can induce autophagy in embryonic stem cells by activating the AMPK pathway and the concomitant suppression of the mTORC1 signaling cascade [66]. Spermidine, which has a neuroprotective effect in patients with cerebral ischemia [67,68], can be found in a variety of foods [67,68]. Spermidine may also exert its beneficial effects with enhanced autophagy through the AMPK-mTOR pathway [69] and, thus, autophagy induction [70]. Spermidine could activate autophagy via the inhibition of mTORC1 [71]. Urolithin A is a natural polyphenol made by gut microbiota [72], which could induce autophagy for cell protection against kidney injury [73]. In addition, Urolithin A also protects against Parkinson’s disease by enhancement of neuronal survival [74]. Urolithin A could induce mitophagy, which may be required for its neuroprotective effect [75]. Urolithin A could increase the phosphorylation level of AKT and mTOR [76]. Anthocyanins, common plant pigments, can activate autophagy and protect neuronal cells [77], which could improve the memory and motor performance of patients with neurodegeneration [78]. Anthocyanins could induce autophagy via the AMPK-mTOR signaling pathways [79]. Diallyl trisulfide is found in garlic oil, which has a neuroprotective effect against ALS model mice [80]. Diallyl trisulfide could decrease the PI3 K/mTOR signaling pathway and increase the expression of AMPK/TSC2 in Hep G2 cells [81], which might result in inducing autophagy and/or suppressing the levels of ROS [82]. Ginkgolic acid has an anti-cancer effect on colon cancer, triggering apoptosis and autophagy by controlling ROS production, in which mTORC1, p-mTOR, and p-S6 kinase could be dose-dependently reduced by the ginkgolic acid [83]. In addition, autophagy inhibitors could block ginkgolic acid-dependent clearance of α-synuclein in Parkinson’s disease [84]. Genistein, the primary isoflavone from soy products, enhances antioxidant enzyme activities and could activate AMPK signaling through the downregulation of mTOR [85]. The SIRT1 (Sirtuin1)/AMPK pathway, combined with inhibiting mTOR signaling, has also been involved in accelerating autophagy by genistein [86], suggesting that genistein-dependent autophagy can diminish cellular senescence (Figure 2).

In view of the fact that more and more various phytochemicals could be applied to the treatment of ALS, it is necessary to have a more comprehensive understanding of the effects and/or potential mechanisms of the phytochemicals on autophagy and/or ALS. In addition, more research should focus on the regulatory mechanisms of mitophagy in ALS and further explore the potential of targeting mitophagy with certain phytochemicals for the prevention and/or treatment of ALS. The use of such compounds could be an opening point for the new therapy of ALS.

## 5. Current Therapeutic Possibilities and Limitations for ALS

With no current cure for the disease, ALS therapeutics seem to have been placed around attempts to slow the progression of the disease and provide symptomatic treatments to maintain patient quality of life (QOL). Therapeutic exercise and/or rehabilitation are also recommended for patients to slow symptomatic progression [87]. Furthermore, multidisciplinary therapy teams are known to improve patient QOL and have the potential to prolong patient survival [88]. However, there is still no cure for ALS that could reverse the progression of the disorder. At present, riluzole and edaravone may be two major disease-modifying drugs for the treatment of ALS [89,90]. The most widely-used drug showing a slight beneficial effect on patient survival [91], riluzole, might have a complex mechanism of biochemical action [92]. Riluzole may increase the survival of ALS patients by up to 18 months [93]. In the experimental study, enhanced mTOR levels and/or attenuated autophagic activity could increase the survival of motor neurons in a dose-dependent manner, suggesting that down-regulation of autophagy might be proffered as a therapeutic procedure for the treatment of ALS [53,94]. Riluzole could exhibit antioxidant capabilities against oxidative, but not nitrosative, stress [95]. Another drug, edaravone, is also an antioxidant compound anticipated to reduce oxidative stress and eliminate lipid peroxidation [96]. Edaravone has been described as having a therapeutic effect in ALS patients, exhibiting reduced functional loss in several neurons [97]. Edaravone has been shown to remove peroxide and/or hydroxyl radicals protecting neurons in ALS [98]. Edaravone could also reduce excessive ROS, s a free radical scavenger, o prevent brain damage [99]. A somewhat unsatisfying efficacy and inconsistency in the potential mechanisms of these conventional drugs may indicate that new strategies are immediately needed to realize therapeutic development for the treatment of ALS. Since emerging evidence has supported the notion that dysregulation of autophagy is critical for the pathogenesis of ALS, the autophagic signal pathway may be a potential and/or crucial therapeutic target [100]. New therapeutic strategies for the ALS community are immediately necessary to combat the exponentially rising epidemiology of this disease [101].

## 6. Next Perspectives

With a complex etiology and no current cure for ALS, broadening the understanding of disease pathology is required to progress with patient care [102]. In general, mitophagy selectively degrades damaged mitochondria to suppress damaged mitochondria-derived ROS that would damage healthy mitochondria and ultimately result in mitochondrial dysfunction. As a major mechanism of mitochondrial quality control, mitophagy could degrade dysfunctional mitochondria to maintain mitochondrial integrity and function. Therefore, activated and/or appropriate mitophagy would prevent ROS from triggering oxidative stresses and inflammatory responses. In particular, counteraction of the process of oxidative stresses may be promising for prolonging life with ALS [103]. Looking for natural compounds with mitophagic actions would provide new insights into the therapeutic intervention for mitochondrial dysfunction-related diseases, including ALS. However, the therapeutic potential of autophagy modulation has not been fully exploited.

Additional efforts are being conducted to develop novel compounds with improved specificity and potency, in which a higher susceptibility of ALS lysosomes compared to healthy control could be suggested [104]. Additional studies and detailed mechanistic insights are also required to fully elucidate and decipher the function of autophagy in the pathogenesis of ALS. Another relevant and promising field of application may be various neurodegenerative diseases or aging-related disorders. The widespread inclusion of natural molecules in functional foods deserves consideration, as it might contribute significantly to the prevention and treatment of ALS, as well as to the improvement of public health. Furthermore, no curative therapeutic approach has been found to date, so further studies should be required to cure these lethal diseases one day.

## Data Availability

Not applicable.

## Abbreviations

ALS: amyotrophic lateral sclerosis	
ATP: adenosine triphosphate	
DNA: deoxyribonucleic acid	
HDAC6; histone deacetylase 6	
mTOR: mechanistic/mammalian target of rapamycin	
MFN1: mitofusin 1	
mTORC: mechanistic/mammalian target of rapamycin complex	
NDP52: nuclear dot protein 52	
PARL: presenilin	associated rhomboid
like	
OPTN: optineurin	
PDK1: 3	phosphoinositide-dependent protein kinase
1	
PINK1: PTEN	induced kinase 1
PTEN: phosphatase and tensin homolog	
QOL: quality of life	
ROS: reactive oxygen species	
SIRT1: Sirtuin 1	
TBK1: TANK	binding kinase 1
TSC2: tuberous sclerosis protein 2	
VDAC1: voltage	dependent anion channel 1
Ub: ubiquitin	
